# AN EXAMINATION OF GENDER DIFFERENCES IN THE PERCEPTION OF ELDERSPEAK

**DOI:** 10.1093/geroni/igad104.2693

**Published:** 2023-12-21

**Authors:** Abby Teply, Hanna Schultz, Jeffrey Buchanan

**Affiliations:** University of Wyoming, Laramie, Wyoming, United States; Minnesota State University, Mankato, Mankato, Minnesota, United States; Minnesota State University, Mankato, Mankato, Minnesota, United States

## Abstract

This study aimed to examine how gender impacts perceptions of elderspeak. Participants (n = 81; ages 65-93) were presented with one of two vignettes set in an assisted living facility to simulate the experience of being a recipient of elderspeak. The vignettes were identical except that the gender of the nursing assistant (NA) differed across vignettes. After reading the vignette, participants were asked to rate their emotional reactions using an adapted version of the Positive and Negative Affect Schedule (Watson et al., 1988) and rate their perceptions of the NA using the Emotional Tone Rating Scale (Williams et al., 2012). Because the goal of the study was to determine possible interactions between the gender of participants and the gender of the NA in the vignette, a two-way MANOVA was utilized. Results indicated a difference in positive and negative affect scores across vignette conditions. Specifically, there was evidence that participants reported lower positive affect and greater negative affect when elderspeak was used by a male NA compared to a female NA. Results suggest that both older men and women perceive elderspeak more negatively when it is used by a male compared to a female. However, elderspeak is not received well, regardless of who uses it, suggesting a need for a nursing assistant training curriculum focused on person-centered communication. Future research should include a more diverse sample in terms of ethnic identity, cultural background, sexual orientation, and gender identity to determine whether these results generalize to a larger population of older adults.

